# Supplementary material for: Macroporous Oil-Sorbents with a High Absorption Capacity and High-Temperature Tolerance Prepared Through Cryo-Polymerization
Source: Polymers (Basel). 2019 Oct 7;11(10):1620. doi: 10.3390/polym11101620 (PMC6835346; doi:10.3390/polym11101620)
Supplement: Supplementary file 1 [file polymers-11-01620-s001.pdf]

polymers

MDPI

# Macroporous Oil-sorbents with High Absorption Capacity and High-temperature Tolerance Prepared through Cryo-polymerization

**Abdul Haleem, Jia-Yun Wang, Hui-Juan Li, Chuan-Shan Hu, Xi-Chuan Li and Wei-Dong He ***

## 1. Nitrogen Adsorption-Desorption Analysis of the Obtained Cryogels

Nitrogen adsorption-desorption technique is widely adopted to determine the porosity and total surface area of micro/meso-porous materials. As shown in Figure S1, the nitrogen adsorption-desorption isotherms of four cryogels are characteristic of Type II curve with very little hysteresis cycle, suggesting that all the cryogels are macro-porous.

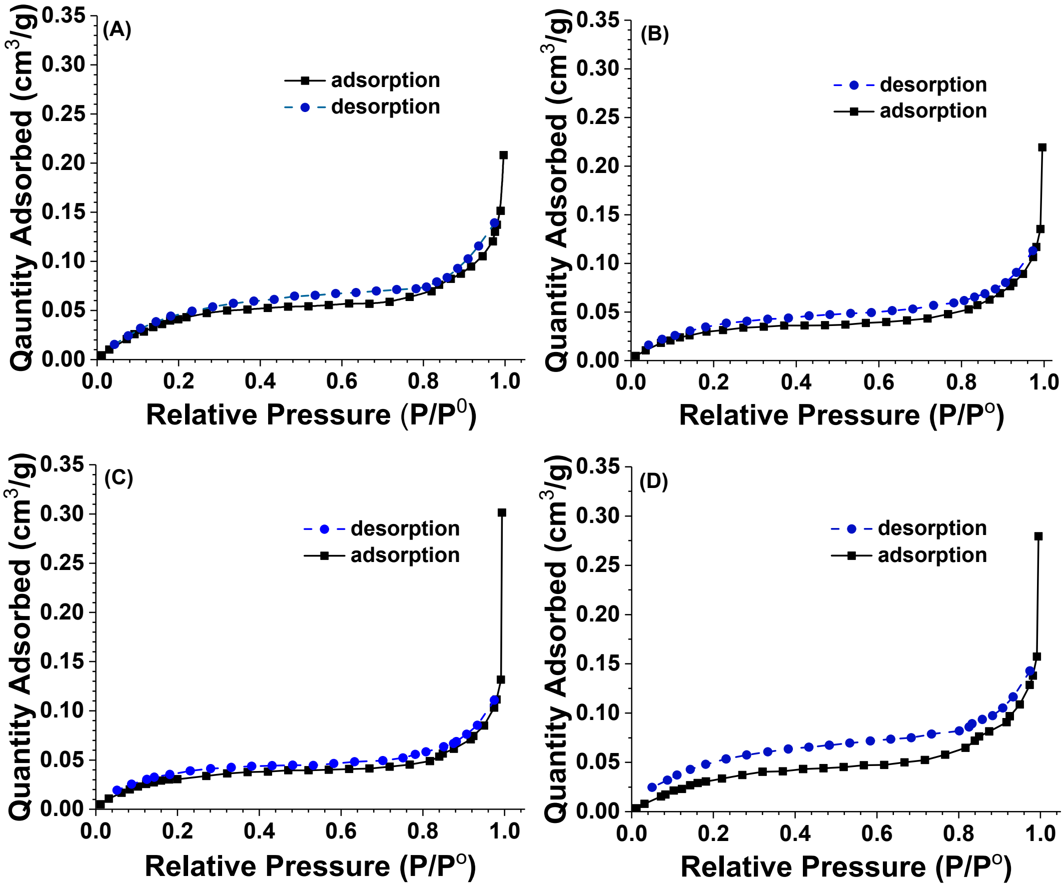


**Figure S1.** Nitrogen adsorption-desorption isotherms of different cryogels (**A**: C-63p2; **B**: C-65p2; **C**: C-67p2; **D**: C-63p4).

## 2. SEM Observation of Cryogels after Heating Treatment at Different Temperatures

The cryogels were kept at different temperatures (25, 50, 100, 150 and 200 °C) in a vacuum oven for 3 h. The inner morphology was observed under SEM. As suggested by Figure S2 (50, 100 and 150 °C), the inner morphology of cryogels treated at lower temperatures hardly changed. However, as shown in Figure S2 (200 °C), C-63p4 cryogel after thermal treatment at 200 °C for 3 h, the less open pores are observed.

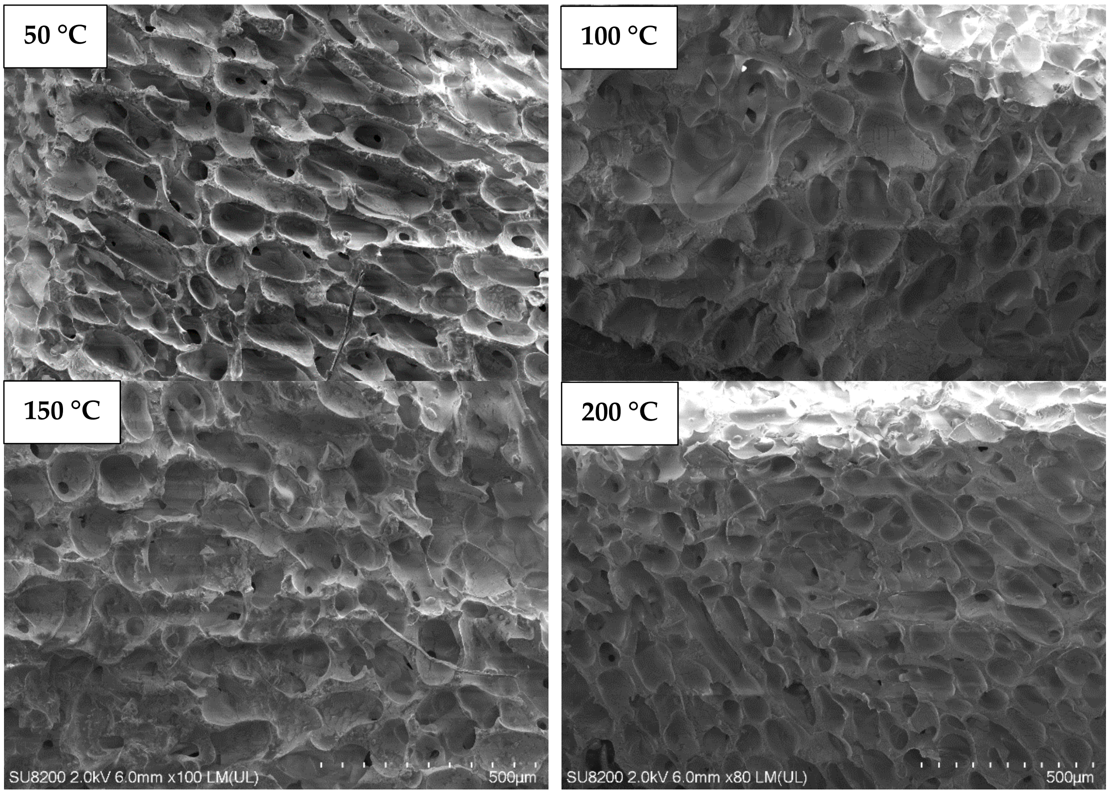


**Figure 2.** Morphology of hydrophobic cryogels fabricated at 4 °C after temperature treatment.

## 3. SEM Observation of Cryogels after Heating Treatment at Different Temperatures

A video for the cryogel to absorb toluene containing Sudan Red was recorded. It shows that the cryogel absorb toluene very quieckly and saturated swelling reaches within 15 s.
